# Use of the Vula App to refer patients in the West Coast District: A descriptive exploratory qualitative study

**DOI:** 10.4102/safp.v64i1.5491

**Published:** 2022-04-25

**Authors:** Louwrens Steyn, Robert J. Mash, Gavin Hendricks

**Affiliations:** 1 Division of Family Medicine and Primary Care, Faculty of Medicine and Health Sciences, Stellenbosch University, Cape Town, South Africa

**Keywords:** primary healthcare, primary care, family medicine, coordination, referral pathways, technology

## Abstract

**Background:**

Referral systems play a pivotal role in coordination and quality of care and should be evaluated for their utility. The Vula App is used by various disciplines and hospitals in South Africa to refer patients. The aim was to explore the perceptions of medical practitioners regarding the use of the Vula App in the West Coast District.

**Methods:**

A descriptive, exploratory qualitative study used semi-structured interviews with 11 medical practitioners. The highest and lowest users of the Vula App were selected from seven district hospitals. Qualitative data analysis used the framework method and Atlas-ti.

**Results:**

There were five themes: impact on the referral process, quality of care, coordination of care, continuous professional development, and how to improve the Vula App. Its use was well established in the outpatient and semi-urgent setting, but participants were hesitant to rely on it for immediate advice. Specialist advice via the Vula App enabled practitioners to manage patients remotely. The referral hand-over function had a positive impact on the coordination of care. Advice and feedback via the Vula App assisted with continuous professional development.

**Conclusion:**

The Vula App is a useful tool to refer patients to the emergency centre and outpatient departments. It can improve the immediate quality of care and sequential coordination of care. It has the potential to enable continuous professional development. There is a need to standardise its use, to ensure electronic information flows back to the district and to integrate the data into the district’s health information system.

## Introduction

The way we practise medicine is being revolutionised by telemedicine and mobile health (m-Health).^1^ The use of smartphone and tablet applications (apps) within healthcare is rapidly expanding,^2^ and it has become socially and professionally acceptable to make use of this technology.^3^ A study performed in the United Kingdom showed that more than 90% of doctors and nurses own smartphones and almost 90% of doctors and 67% of nurses were using communication apps as part of their clinical practice.^4^ The dawn of m-Health reveals a new horizon of clinical communication through tools such as Line, WeChat, Viber and WhatsApp.^1^ In South Africa, a medical communication app that has started to gain significant momentum is Vula Mobile.^5^ Vula Mobile is a smartphone app that is designed to provide quick access to specialist advice and provide an efficient way to refer patients between generalist and specialist care.^5^

Incomplete, fragmented and disorganised clinical communication contributes to the bulk of medical errors during patient management.^6^ Traditional methods of communications that rely on face-to-face meetings, telephone conversations and alphanumeric paging systems all report adverse events with patient care.^6^ The mounting complexity of patient data with the increasing burden of patient numbers, demands efficient and effective communication between healthcare providers, to ensure a smooth-running health service.^7^

The lack of seamless access to patient information across the care pathway remains one of the most pressing issues in healthcare.^8^ The absence of an integrated system poses a threat to the quality of care and patient safety.^9^ Utilising smartphone apps to overcome this is a possible solution, but it can also have challenges, such as secure access to patient data and patient confidentiality.^7,9^ A recent study at the University Hospital Limerick in Ireland revealed that 97% of the surveyed doctors shared sensitive information over instant messenger technology without patient consent, despite 68% of them being concerned about the distribution of this information.^10^ The Health Professions Council of South Africa (HPCSA) has ethical guidelines to guide healthcare providers on the use of telemedicine but not for social media.^11^ However, a practical and ethical guide for medical students and doctors was published by the South African Medical Association (SAMA).^12^

Effective and efficient communication is the key to safe and high-quality patient care.^13^ In hospital, communication challenges include large multidisciplinary teams with complex hierarchies guiding patient care, a proliferation of clinical information that is often time critical and the necessity of staff travel within hospitals and between healthcare sites.^13^ The use of communication apps on mobile phones to communicate with colleagues is fast, efficient, portable and convenient.^14^ These apps are often free, easily available and in widespread use.^13^ This facilitates rapid communication within clinical teams and closed groups, overview of clinical care and increased involvement by senior clinicians, an enhanced handover process between clinicians, easy communication of patient results and rapid changes to patient management plans.^2^ With multiple healthcare staff caring for patients, enhanced communication supports greater efficiency and better outcomes.^15^ Smartphone communication apps, therefore, facilitate communication between healthcare workers to improve health outcomes for patients.^13^

Vula Mobile has been used in South Africa to refer patients since 2014.^5^ Although it was designed for ophthalmology referrals, Vula has since been expanded to over 16 specialities, each with its own custom-designed referral template. It has several advantages over traditional phone-based referrals in that it is more structured and ensures that relevant information and images are shared to allow for more informed clinical decision-making. It also has potential for feedback and upskilling of the referring healthcare workers.^16^ Personal smartphone use may also be more convenient than landlines and improve response time from the registrar on call.^16^ A recent study conducted by the Orthopaedic Department of Tygerberg hospital suggests that the Vula App is a successful alternative to traditional referral methods.^17^ Furthermore, the finding that one-third of referrals were managed by giving advice over the app suggests that the Vula App can decrease the workload of overburdened referral hospitals.^17^

In the future, electronic and mobile systems will play pivotal roles in healthcare delivery.^9^ Healthcare and academic institutions should support the use of technology and not stifle technological progress. However, the drive for the development of apps needs to be supported by robust governance frameworks, evaluation of the clinical outcomes and potential unintended consequences.^9^ The ability of the Vula App to strengthen the referral system in the public sector and upskill referring doctors should be further explored.^17^

The aim was to explore the perceptions of medical practitioners regarding the use of the VulaApp to refer patients in the West Coast District of the Western Cape to specialist hospital-based care. Specific objectives included the following:

- To explore the impact on the referral process.
- To explore the impact on quality of care.
- To explore the impact on coordination of care between district and regional/tertiary facilities.
- To explore the impact on continuous professional development.
- To explore any technical and/or practical challenges and possible ways to improve the Vula App.

## Research methods and design

### Study design

A qualitative, descriptive, exploratory study was conducted using semi-structured interviews with participants who were purposefully selected.

### Setting

This study was conducted in the district hospitals of the West Coast District in the Western Cape province of South Africa. These hospitals were supervised by a medical or clinical manager, a family physician or a senior medical officer (MO). The remaining staff complement was made up of registrars, MOs with varying years of experience and doctors doing their community service.

The West Coast district was made up of the following sub-districts: Bergrivier, Cederberg, Matzikama, Saldanha Bay and Swartland. There was a total of seven district hospitals providing healthcare: Citrusdal Hospital, Clanwilliam Hospital, Lapa Munnik Hospital (Porterville), Radie Kotze Hospital (Piketberg), Swartland Hospital (Malmesbury), Vredenburg Hospital and Vredendal Hospital.

Patients were referred from these district hospitals to a regional or tertiary hospital. Regional hospitals were Paarl Hospital and New Somerset Hospital, and tertiary hospitals were Tygerberg Hospital, Groote Schuur Hospital and Red Cross War Memorial Children’s Hospital.

To refer a patient to one of these hospitals, you had to speak with the doctor on call for the medical speciality you would like to refer your patient to. This communication with the doctor on-call could be via telephone, e-mail, WhatsApp, text messaging or the Vula App.

### Study population

The study population included all medical practitioners who were clinically active at the district hospitals in the West Coast District. The participants must have used the Vula App. Locum and visiting medical practitioners, who were not employed in the West Coast district, were excluded.

### Sample size and sampling

Fourteen information-rich participants were purposefully selected. Purposeful selection was done by means of maximal variation sampling, according to low and high Vula App utilisation. The administrators of Vula provided a list of doctors that were registered Vula users at each district hospital and the number of Vula referrals they had made. The lowest (participant that sent the lowest number of Vula referrals since registration as a Vula user) and highest (participant that sent the highest number of Vula referrals since registration as a Vula user) user from each of the seven hospitals were selected. These 14 purposefully selected participants were invited to partake in the study. The last three interviews were reviewed to determine whether data saturation was reached, and further interviews could be conducted if necessary.

### Data collection

A semi-structured interview guide was developed by L.S. with input from R.J.M. based on issues from the literature. Two pilot interviews were done by L.S. with a clinical manager and family physician from the West Coast District who were not invited to participate in the study. These interviews were analysed by L.S. and R.J.M. and the interview guide was then further refined. The semi-structured interview guide was internally validated for use in this study. The interview guide consisted of an opening question followed by a set of topics that could be explored by asking further open-ended questions. Demographic data were also collected to describe the participants and included the gender, age, position held and years of experience. The opening question was as follows: ‘How would you describe your experience of using Vula to refer patients from your facility to a regional or tertiary hospital?’

The topics that were explored included the impact of the Vula App on the referral process, quality of care, coordination of care, continuous professional development, technical and/or practical issues with the app and possible ways on how to improve the app.

Semi-structured interviews were conducted by L.S. either face-to-face or virtually via Zoom and no one else was present during the interviews. Appointments for the interviews were made well in advance to give the participants time to make the necessary arrangements to be available and free of any clinical duties during the time of the interview. The interviews were conducted in English or Afrikaans per the participant’s preference. The researcher was able to conduct the interviews in both English and Afrikaans and no translator was required. Except for the virtual interviews, the interviews were conducted at the workplace of the participant. The interviews lasted between 15 min and 60 min. The researcher received training in communication and qualitative interviewing in preparation for the data collection of this study. The interviews were audio-recorded, and the researcher made field notes of his further observations. The interviewer made use of a variety of communication skills such as open questions, reflective listening, clarifying and summarising.

As a registrar in family medicine, who worked in the West Coast District, L.S. used the Vula App and had to conduct interviews with his colleagues at local district hospitals. Participants were aware that he was performing this research for his Master of Medicine degree. He was aware that he needed to remain neutral, set aside his own views and perceptions of the app and needed to listen to the participants from the perspective of a researcher. L.S. received training in qualitative methods and interviewing from Stellenbosch University but had no prior experience as a qualitative researcher.

### Data analysis

The interviews were transcribed verbatim and before thematic analysis the transcripts were checked for accuracy and any mistakes corrected against the original audio recording. The researcher used member checking to confirm the accuracy of the data. The researcher performed the data analysis with the assistance of ATLAS.ti software (version 8). The framework method of qualitative data analysis was used to analyse the transcriptions.^18^ The following steps were used:

- Familiarisation: The researcher familiarised himself with the transcripts and identified key issues and ideas emerging from the data that could be coded.
- Developing a coding index: The researcher identified and defined codes to be used and organised them into categories.
- Indexing: The researcher applied codes systematically to all qualitative data.
- Charting: The researcher re-arranged the data into a series of charts, bringing all the data with the same code and in the same category together.
- Interpretation: The researcher read each chart and interpreted the data in order to identify key themes. The range and nature of ideas and experiences in each theme were described and interpreted.

### Trustworthiness

The analysis of the data was supervised by R.J.M., particularly the construction of the coding index and the interpretation of the findings. The analysis followed a clear stepwise process that could be audited with the help of Atlas.ti. The study setting and participants are described in as much detail as possible to enable readers to make decisions on the transferability of the findings. The researcher was mindful of his own reactions during the interviews and the data analysis to remain conscious of his own assumptions and beliefs that might influence the interpretation.

## Findings

Eleven participants were interviewed as three of the 14 who were invited, declined consent. Ten interviews were in person, and one interview was via Zoom. Three of the medical practitioners were also managers, five were permanent MOs, and three were community service MOs. Seven participants were male and four were female. Participants’ ages ranged from 27 to 63 years. All participants were registered VulaApp users, six were high volume users and five were low volume users.

The findings are presented under five main themes:

- Perceptions of impact on the referral process
- Perceptions of impact on quality of care
- Perceptions of impact on coordination of care
- Perceptions of impact on continuous professional development
- Perceptions on how to improve the Vula App.

### Perceptions of impact on the referral process

Participants had a mixed overall experience with the VulaApp. They felt that the concept was good, but the actual utility had not met expectations. The app was operator dependent, and the responsiveness and quality of the responses were dependent on the person you sent your referral to. Good-quality cell phone reception and lack of mobile data were also mentioned as factors influencing the usage of the app:

‘It’s very user-dependent and very dependent on who is on the other end. Some of the responses have been quite well and quite fast and sometimes you wait an hour or two or three before a response.’ (27 years old, male, community service medical officer [Cosmo])

‘The App itself works well, but signal reception can be a problem.’ (34 years old, male, MO)

Participants had good experiences using the app in non-urgent outpatient settings but found it frustrating and time-consuming in the emergency setting. In particular, the trauma department at Tygerberg Hospital used Vula App as the preferred method of referral and the participants’ experience was that this often caused a delay and anxiety if you needed immediate advice:

‘In an emergency, a telephone call is always a better option. Specifically if you need someone quickly.’ (28 years old, female, Cosmo)

Vula App was not used by all departments at referral hospitals and participants needed to know the preferred method of communication. Furthermore, some departments wanted elective referrals to be done via Vula App and urgent referrals via telephone. Other departments wanted all referrals to be discussed telephonically first and then a Vula App referral was sent with all the detailed information. In the departments that used Vula App as the preferred method of communication, participants highlighted that it put you directly in contact with the specialist on call and gave you the option to call the specific person. This was a time-saving feature in comparison to a telephone call via a hospital switchboard, which often was a slow and frustrating process:

‘So what I like about the App is it’s quite handy, it’s always available. I think most of us have our cellphones with us 24 hours a day. And what’s nice is you have access to a specialist.’ (28 years old, female, Cosmo)

Departments were also responsible for the design of the referral template on the app that referring practitioners should complete. Departments like ear-nose-and-throat (ENT), ophthalmology, dermatology and the burns unit had customised these templates so that they were easier to use and concisely gave all the necessary information. In other departments, the default template was blank and the referring healthcare worker needed to decide what information to give. This often led to multiple questions from the accepting practitioner and required back-and-forth communication:

‘What’s also nice is a lot of the departments have now specialized their Vula. So we have things for example Ophthalmology has their built-in eye test, ENT has the built-in hearing test, and it makes it a lot easier to do the referrals. So the specialist makes it very clear exactly what they want and how they want it.’ (28 years old, female, Cosmo)

Participants felt there was a place for different ways of communication on the service delivery platform. A telephone call was the preferred way of communication in an emergency as it was instant and one could get immediate advice. The negative aspect of a telephone call was that it could be difficult to get hold of the person via the hospital switchboard and the person might also be busy and not available at that moment. Participants preferred to use email for non-urgent communication, for example, booking elective procedures and providing medical reports:

‘I would like to discuss or refer the patient, I pick up the phone, phone them and then I have an answer.’ (30 years old, female, MO)

Participants preferred using Vula App to refer semi-urgent and elective patients to specialist outpatient clinics. The app allowed you to send photos, ask for advice on ongoing management or arrange for the patient to be seen at a specialist clinic. Referral details were also saved in the app, which allowed practitioners to review the same patient at a later date. The ‘chat’ function of the app helped to clarify arrangements, allowed for ongoing discussion of patient management and could be used to give feedback to the referring practitioner:

‘So I think in an OPD [*outpatien t department*] sense I think Vula is okay to use because then it’s not an emergency – it can take its time in terms of getting hold of the person and getting any sort of feedback.’ (27 years old, male, Cosmo)

Participants were also positive about WhatsApp as an alternative method of communication, because it was used by almost everyone, was user friendly, quick and convenient; could include photos, videos and voice notes and could provide a quick answer to a question:

‘I prefer WhatsApp because it’s easy, it’s functional and sometimes WhatsApp is faster.’ (28 years old,female, Cosmo)

‘WhatsApp sort of created a faster way of chatting to a person and getting an answer faster.’ (34 years old,male, MO)

Participants, however, expressed their concern about patient confidentiality when discussing patients via a chat-based app. There was a particular concern with WhatsApp and where the information was being stored. In terms of the safety of patient information, participants felt that the VulaApp was the better option:

‘So I know WhatsApp is not too clear, I know the Department of Health doesn’t allow WhatsApp. They’ve got a lot of concerns about it and there have been countless emails about how we shouldn’t do it, it’s not so clear. Images can be leaked, it can be hacked.’ (28 years old, female, Cosmo)

### Perceptions of impact on quality of care

The greatest attribute of the app was that it could put a practitioner from a rural district hospital in contact with a specialist. This enabled the practitioner to manage the patient better in remote or rural settings. By being able to manage the patient at their local facility, unnecessary specialist outpatient appointments were avoided and the limited transport capacity was not overburdened. This allowed for a win–win situation where the patient obtained immediate specialist advice and the healthcare resources were used more efficiently:

‘It is fantastic to be able to practice medicine at any place and time and be able to contact a specialist directly if you have a smartphone with the Vula Mobile App installed and a Wifi or 3G connection.’ (64 years old, male, Medical Manager)

The custom-made templates also prompted the referring practitioner to properly examine and investigate their patients. If you could not supply all the information needed to complete the referral, then you needed to go back to the patient to confirm or exclude whatever was missed:

‘Some departments have their own referral template which asks for specific information, this makes referrals easier.’ (28 years old, female, Cosmo)

Timely and useful advice on patient management by the accepting practitioner, before the transfer of the patient, could significantly improve patient care and ensure that a properly stabilised patient was transferred:

‘The doctor on the other end can advise me on what needs to be done before I transfer the patient.’ (64 years old, male, Medical Manager)

### Perceptions of impact on coordination of care

The Vula App linked all levels of care together, from primary healthcare to district, regional and tertiary hospitals. A referral could be sent from any of these levels to another. Referrals were usually from a lower level of care to a higher level of care. However, the patient was not referred back to the initial facility via the app. Participants thought that if this was done it would substantially improve the coordination of care:

‘It will be great if they refer the patient back to us for further care via the Vula Mobile App.’ (34 years old, male, MO)

Practitioners were often not aware that clinical management at a higher level of care had been completed and that the patient could be seen again at their local facility. Such feedback should include the final diagnosis and management in-hospital, the current management that needed to be continued, and what should be done in the future for the patient.

A recent change in the app that improved coordination of care was the ‘hand-over function’. This function enabled a practitioner to hand over a referral that was not completed to another colleague who took over the management of the patient. This function worked extremely well for coordination between shifts as all open referrals were handed over on the app to the new team on duty.

A further improvement that would have a positive impact on coordination of care would be if the app allowed you to refer the same patient to more than one department simultaneously. It was necessary to complete a new referral on the app for each department, which was time consuming and duplicated information:

‘So the handover function that came in recently, where I can hand over a patient to one of my colleagues, or in Tygerberg trauma, they’ll hand it over to the next shift. That works very well. But it would be great if that same function was used to refer to another department.’ (36 years old, male, MO)

‘It would be lovely to add a second or a third person because sometimes we do have patients for example who are poly traumas that usually want multiple departments involved but no, it doesn’t offer that. You have to actually refer to every department individually.’ (28 years old, female, Cosmo)

### Perceptions of impact on continuous professional development

The general experience among participants was that Vula App had a limited impact on continuous professional development. Participants emphasised the importance of good quality advice and feedback on management by the accepting practitioners. Participants mentioned that in some instances they were able to manage a new case without discussion or referral because they have gained the necessary knowledge from previous referrals. Participants also felt that academic resources within the app or links to resources could be helpful to manage patients and expand one’s knowledge.

Some of the participants had gone back to their previous VulaApp referrals and reflected on what went well, what did not go well, and what could be improved in the future. Old referrals had also been used to write up case studies and do case presentations at local academic meetings:

‘I think for myself it has been very helpful so far and it’s nice. You can see that you have referred your patient, you get the response and you have it there with you, what is the plan for your patient, etc., etc. and like I say, you can always go back to it because you have it stored on your phone.’ (28 years old, female, Cosmo)

From the medical managers’ points of view, they suggested that the Vula App statistics on referrals made from their facilities could be used as part of the monthly morbidity and mortality meeting. These statistics could help to describe the burden of disease at a specific facility:

‘It would be great if we could use that information to see what sort of stuff is going to Tygerberg trauma, how many head injuries are we seeing in the periphery? Serious head injuries, you know, all of that stuff will obviously show. So that it can be used, it can be useful for epidemiology purposes I think.’ (36 years old, male, MO)

### Perceptions on how to improve the Vula App

Participants felt that the more the app is used and the more departments that endorse the app as their preferred method of communication, the better referral via Vula App becomes. They also suggested that departments should have a dedicated ‘doctor on call’ to respond to Vula App referrals to improve response times. Training on how to send a referral and how to respond and give feedback via the app would be beneficial for all. Participants would like to send not only photos but also videos via the app[AQ8]:

‘The only thing I have noted lastly is that you can’t actually upload any videos to the App and sometimes some of the doctors would like videos of the patient.’ (27 years old, male, Cosmo)

A desktop personal computer version of the app, available in healthcare facilities, would also be useful. If these computers were connected via a network, it would nullify the concern about cell phone reception and the availability of mobile data. Being able to print a referral from the app would also avoid the additional work of having to write out a referral letter to accompany the patient to the accepting facility:

‘I wish you could just print out the Vula as a referral letter and not have to do all that in writing from scratch to refer.’ (30 years old, female, MO)

A further improvement that was suggested was a notification system to inform you if your referral is pending, has been delivered, or has been read:

‘So I think what may be another suggestion to improve the App maybe would then be to have sort of a notification, just when the doctor on the other side opens your referral so that you know the referral has been read, because that’s sort of, otherwise you wait for them to message in the chat and then you have to say okay, now I know that you have seen my referral, otherwise you don’t know whether they have seen it yet or not.’ (28 years old, female, Cosmo)

In addition, a practitioner cannot update their personal particulars within the app. If you move from one facility to another, you need to inform a Vula App administrator about your new place of work to update your details, which can take several days. The inability to change your place of work was particularly hampering to practitioners working at more than one facility:

‘I have actually had the App since I was an intern, and I did internship at Groote Schuur. So what I have noticed is that you actually can’t change your location, to change your location from where you have registered, you have to e-mail Vula.’ (28 years old, female, Cosmo)

## Discussion

**FIGURE 1:**
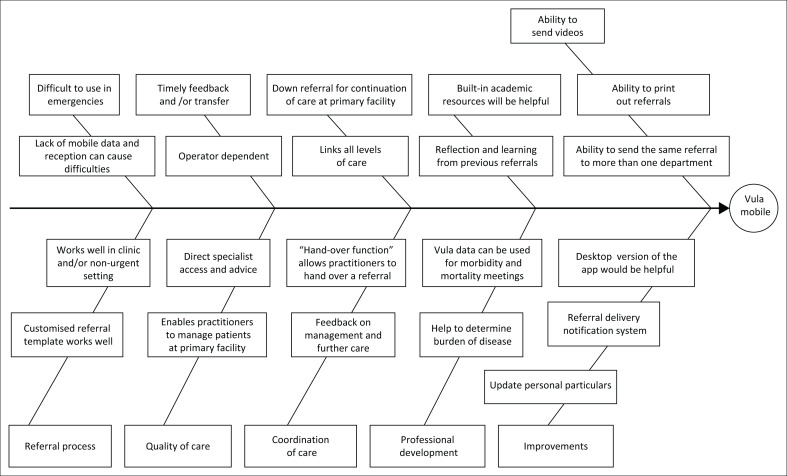
Fishbone diagram summarising key findings.

Figure 1 presents the key findings of the study in a conceptual framework. The key findings have been organised into five main categories: impact on the referral process, quality of care, coordination of care, continuous professional development and how to improve the Vula App.

### Referral process

Vula App has established itself as a successful alternative to traditional referral processes.^17^ It is in widespread use across the healthcare platform and has more than 20 000 users.^5^ The app was most useful in organising emergency and out-patient referrals although a phone call was still preferred for immediate communication in very urgent emergencies. Even in these cases, the app could assist by providing the cellphone number of the doctor-on-call and avoiding the hospital switchboard. A recent study found that Vula played a significant role in decreasing the number of non-urgent ‘green’ patients being referred to the emergency unit from primary care.^19^ The study, however, did not mention response times and how quickly urgent emergency referrals were attended to. The place of the app in the referral system should be evaluated and a decision needs to be made as to how the app is best utilised. Participants in this study felt that the app worked well in the out-patient setting and the non-life-threatening emergency setting. At the time of this study, there was no consistent policy in place regarding the use of Vula App. Specialist departments can decide individually if they want to adopt Vula and how they use it. A policy on the use of the Vula App for referrals by all specialist departments will greatly benefit the referral process. This policy will enable practitioners to know when and how to use the Vula App for maximum efficiency. Vula App is compliant with the latest Protection of Personal Information (POPI) legislation and therefore provides practitioners with a more secure platform to share patient information than other apps.^5^

The app allows healthcare workers to be contacted directly as their cellphone numbers are available in the app; this avoids delays and time being wasted by using hospital switchboards.^20^ The app uses asynchronous communication, which is more convenient than synchronous; however, it may lead to a delay in patient management or transfer if either user does not respond promptly.^20^

### Quality of care

Vula App can have a significant impact on the quality of care in the district health services although this relies on constructive and timely feedback to improve ongoing or transitional care before transfer. The value of outreach from specialists to the district health services has been well described.^21^ The Vula App provides a virtual way of providing such outreach when a referral is being considered. In a resource-constrained setting, it is beneficial for both the patient and the healthcare sector if a patient can be managed at the local healthcare facility.^22^ Patient outcomes also significantly improve if the patient is timeously and correctly managed before transfer.^23^ The app assists and prompts practitioners to make good quality and appropriate referrals, but any impact on the quality of care depends on the responding practitioner giving useful and evidence-based advice.

Facilities can also use the Vula App’s data to evaluate the pattern of referrals and morbidity profiles or identify specific cases for morbidity and mortality meetings. These activities will ensure ongoing improvement of quality of care as key areas of concern are identified, addressed and rectified.

### Coordination of care

Sequential coordination of care has been evaluated as poor for both in-patient and out-patient referrals.^24^ Using electronic medical records that are shared between levels of care can improve the availability and transfer of information.^25^ At the moment, South Africa does not have a comprehensive electronic medical record although in the Western Cape the available electronic data on patients is collated in a single patient viewer system. Currently Vula App is not integrated into the single patient viewer system but does have the ability to coordinate care during referrals, reduce inappropriate referrals, standardise the transfer of information and improve quality of care.

Although the app connects one level of care with another, the communication is between two practitioners and is not necessarily captured in the medical record. In addition, the communication is almost entirely one-sided, from the referring doctor to the receiving doctor, with little information flowing back once the patient is seen or discharged. The Western Cape has also developed an electronic discharge system for hospital in-patients, the Electronic Continuity of Care Record (ECCR), which can be accessed by doctors in the district. This could be extended to provide information on patients seen in the emergency centre or out-patients. The Vula App could provide feedback, but is less structured and the information would only go to the referring doctor. There is a need to ensure adequate transfer of information from the referral hospital to the district to ensure coordination of care.

Previous studies have shown that the app improves relationships between doctors at different levels of the health system, with more trust and respect, as well as a greater understanding of each other’s contexts.^17,25^ Practitioners should be encouraged to give each other feedback and change the culture in the government sector of minimal or no feedback to primary care. Good-quality feedback, especially when a patient is referred back to their primary care facility, enables practitioners to continue with the expected care and future management.

### Professional development

A role of Vula App that has been underutilised thus far is the potential to provide continuous professional development. The most important contribution will be in the form of feedback. Healthcare workers desire feedback about the patients they refer.^21^ Feedback is important especially to junior colleagues as it assists them in learning how to manage conditions and enables them to function more independently. Community service doctors are often working in remote, rural settings with no or minimal senior support, and by giving them good quality feedback you are upskilling them to fulfil their role. Good quality constructive feedback includes what was done well and advice on how to improve or do even better in the future. By giving feedback in this manner, reflection and adult learning will be stimulated and this would be beneficial for the practitioner, the patient and the healthcare sector. A platform to earn continuing professional development (CPD) points via the use of the app can also be explored.

### Limitations

Limitations of this study include the following:

- All the participants worked in public sector district hospitals and referred patients using Vula App. The findings might have been different if practitioners that receive referrals, general practitioners in private practice, health managers or information management experts were interviewed.
- Three of the invited participants that were purposefully selected, declined consent. The last three interviews were reviewed and as there were no new themes developed from these interviews, data saturation was reached and no further participants were invited.
- The researcher performed the data analysis alone, but the interpretation of the data and the findings as described were reviewed by his supervisor to enhance their trustworthiness.

The hospitals involved in this study are all small district hospitals within the West Coast District of the Western Cape. The findings of this study might well be transferable to other small district hospitals in the Western Cape and other provinces that are staffed the same way and have similar referral pathways. Findings from the larger district or regional hospitals, with specialist departments that manage their own referrals, might well be different.

### Recommendations

The following recommendations can be made from the findings of this study:

- It would be helpful for policy recommendations to standardise the use of the Vula App across all specialist departments and to clarify what types of referrals best suit the Vula App. It is difficult for the referring practitioner to have a plethora of different rules and expectations from specialist departments.
- There is a need for a system to provide electronic information on patients who are admitted to the referral hospital, seen and discharged from the emergency centre or consulted in the out-patients. The Vula App does not appear ideal for this although feedback on care received in the emergency centre or outpatients could fill a gap in the current system.
- Practitioners that respond to Vula referrals should be encouraged to give good quality feedback as this will improve the immediate care of the patient, benefits coordination of care and potentially provides professional development for the referring practitioner.
- Integrating data from Vula App into the information system may help facilities monitor the referral pathway, determine local morbidity, help with risk management and quality improvement, as well as continuous professional development. Attention should be given to integrating data into the provincial data centre and single patient viewer or allowing facility managers access to reports from the Vula App system.
- A few key improvements will contribute significantly to the utilisation of the app by practitioners. These improvements include the following:
  ■ Provision of good Wi-Fi at healthcare facilities.
  ■ The ability to send videos and voice notes in addition to text and images.
  ■ The ability to send the same referral to more than one specialist department.
  ■ The introduction of a notification system to indicate if a referral is delivered and read.
  ■ A desktop version of the app installed on personal computers in the healthcare facilities.
  ■ The ability to print out a referral from the app.
  ■ To be able to update your personal particulars in the app.

## Conclusion

The Vula App is a useful tool to refer patients to the emergency centre and outpatient departments. Utilisation of the Vula App is well established in the outpatient and semi-urgent emergency centre setting, but further clarification is needed on the use of the Vula App when immediate advice is needed. The Vula App can improve the immediate quality of care by helping practitioners to manage patients adequately before transfer and it enables practitioners to manage patients remotely. It improves sequential coordination of care as patients are referred from district level to specialist level, but the lack of information flowing back to the district is still a missing link in optimal coordination of care. Feedback on patient management can stimulate learning and reflection of the practitioner, but the potential of the Vula App to enable continuing professional development is still underutilised. There is a need to standardise the use of the Vula App, ensure that electronic information flows back to the district and integrate the data into the district health information system.
